# *Bacillus amyloliquefaciens* L-S60 Reforms the Rhizosphere Bacterial Community and Improves Growth Conditions in Cucumber Plug Seedling

**DOI:** 10.3389/fmicb.2017.02620

**Published:** 2017-12-22

**Authors:** Yuxuan Qin, Qingmao Shang, Ying Zhang, Pinglan Li, Yunrong Chai

**Affiliations:** ^1^Beijing Advanced Innovation Center for Food Nutrition and Human Health, College of Food Science and Nutritional Engineering, Key Laboratory of Functional Dairy, China Agricultural University, Beijing, China; ^2^Department of Biology, Northeastern University, Boston, MA, United States; ^3^Key Laboratory of Biology and Genetic Improvement of Horticultural Crops, Institute of Vegetables and Flowers, Chinese Academy of Agricultural Sciences, Beijing, China

**Keywords:** next-generation sequencing, *B. amyloliquefaciens* L-S60, bacterial community, cucumber, plug seedling, growth condition

## Abstract

Vegetable plug seedling has become the most important way to produce vegetable seedlings in China. This seedling method can significantly improve the quality and yield of vegetables compared to conventional methods. In the process of plug seedling, chemical fertilizers or pesticides are often used to improve the yield of the seedlings albeit with increasing concerns. Meanwhile, little is known about the impact of beneficial bacteria on the rhizosphere microbiota and the growth conditions of vegetables during plug seedling. In this study, we applied a culture-independent next-generation sequencing-based approach and investigated the impact of a plant beneficial bacterium, *Bacillus amyloliquefaciens* L-S60, on the composition and dynamics of rhizosphere microbiota and the growth conditions of cucumbers during plug seedling. Our results showed that application of L-S60 significantly altered the structure of the bacterial community associated with the cucumber seedling; presence of beneficial rhizosphere species such as *Bacillus, Rhodanobacter, Paenibacillus, Pseudomonas, Nonomuraea*, and *Agrobacterium* was higher upon L-S60 treatment than in the control group. We also measured the impact of L-S60 application on the physiological properties of the cucumber seedlings as well as the availability of main mineral elements in the seedling at different time points during the plug seedling. Results from those measurements indicated that L-S60 application promoted growth conditions of cucumber seedlings and that more available mineral elements were detected in the cucumber seedlings from the L-S60 treated group than from the control group. The findings in this study provided evidence for the beneficial effects of plant growth-promoting rhizosphere bacteria on the bacterial community composition and growth conditions of the vegetables during plug seedling.

## Introduction

Cucumber (*Cucumis sativus L*.) is one of the major vegetables grown in China. According to the data published by United Nations Food and Agriculture Organization (FAO, http://faostat.fao.org), the area devoted to cucumber cultivation in China has increased annually since 1997 and is currently ranked No. 1 in the world. In 2013, the cultivation area of Chinese cucumber reached 1,166,690 hm^2^ and the total production was 54,362,750 tons. In China, chemical pesticides and fertilizers are still widely used in the field of agriculture to maintain the yield of crops by preventing them from serious crop diseases. Due to increasing concerns about food safety and environmental pollutions, the usage of traditional chemical pesticides and fertilizers has been gradually reduced in the agricultural field. Thus, researchers are now seeking effective alternative solutions to manage crop disease (Alabouvette et al., 2006).

Certain plant root-associated bacteria show beneficial effects on plant growth, and are thus defined as plant growth-promoting rhizobacteria (PGPR) (Kloepper et al., 1981). PGPR antagonize soil pathogens by competing for resources or by producing antibiotics or lytic enzymes (Ali et al., 2014). Currently, PGPR mainly consist of bacteria from the genus *Rhizobium, Azospirillum, Bacillus*, and *Pseudomonas*. Those bacteria are applied widely in the field of agriculture in promoting seedling efficiency, plant biomass, and disease control. Bacteria belonging to the *Bacillus* genus are commonly present in the rhizosphere of plants (Hallmann et al., 1999). *Bacillus* species are known for their capacity to form dormant spores, which allow them to survive harsh environmental conditions (Piggot and Hilbert, 2004). *Bacillus* species also show strong anti-pathogen capacities through the production of non-ribosomal cyclic lipopeptides (Chen et al., 2012; Li et al., 2014; Chowdhury et al., 2015; Zhang et al., 2016). *Bacillus amyloliquefaciens* FZB42 is one of the well-studied and widely applied PGPR. FZB42 has a great capacity to produce a diverse array of antimicrobial compounds. Its antifungal activity is mainly due to non-ribosomal synthesis of the cyclic lipopeptides bacillomycin D and fengycin whilst its antibacterial activity is primarily due to non-ribosomally synthesized polyketides (Chen et al., 2007). *Bacillus amyloliquefaciens* SQR9 is another PGPR strain that shows predominant antagonistic activities against a broad range of soilborne pathogens (Li et al., 2014). In addition, Chen et al. found that environmental isolates of *B. subtills* could control the tomato wilt disease by forming biofilms on the tomato roots (Chen et al., 2012). The current study focuses on *B. amyloliquefaciens* L-S60, a PGPR strain isolated from the turfy soil in Beijing, China (Qin et al., 2015). This strain demonstrates a strong inhibition capacity against several vegetable fungal pathogens, including *Rhizoctonia solani, Fusarium oxysporum*, and *Phytophthora capsici*. It also shows a strong capability in phosphorus and potassium assimilation (Qin et al., 2015).

The roles of rhizosphere microbial communities in plant growth and health have been studied by various types of methods, including both culture-dependent and independent approaches (Kaiser et al., 2001; Hao da et al., 2008; Andreote et al., 2009). However, it is known that only a small proportion of the microorganisms in soil is culturable (Pham and Kim, 2012). Therefore, most culture-dependent approaches show limitation on characterization of microbial communities. Next-Generation Sequencing (NGS) is a new DNA sequencing method, which relies on the detection of pyrophosphate release upon nucleotide incorporation, rather than chain termination with dideoxynucleotides (Shendure and Ji, 2008), and it is a culture-independent method for the environmental microbiota analysis. Comparing to other techniques such as DGGE and T-RFLP, NGS provides more accurate identification of individual species and better insights into the dynamics of the microbial community (Lee et al., 2011). With the development of NGS, more detailed analyses of rhizosphere microbial communities become feasible.

In order to improve the quality and yield, cucumber and many other vegetables' seedlings are now mainly produced by the plug seedling method in China. PGPR has now been increasingly applied in vegetable seedling factories and demonstrated great efficacy in plant growth promotion and disease prevention (Dobbelaere et al., 2003; Guo et al., 2004; Zahir et al., 2004; Saravanakumar et al., 2008). Although studies about the impacts of the PGPR on the rhizosphere microbial communities and the plant growth have been performed using different methods (Ali et al., 2014; Kröber et al., 2014; Chowdhury et al., 2015), research on the impacts of PGPR on rhizosphere microbial communities during the entire plug seedling process of cucumber by means of NGS has not been reported. In this study, we applied NGS and performed experiments to investigate the effects of *B. amyloliquefaciens* L-S60 on the rhizosphere microbiota and the growth conditions of cucumber plug seedling process.

## Materials and methods

### Strain and medium

*Bacillus amyloliquefaciens* L-S60 was isolated from the turfy soil in Beijing, China. The strain was deposited at China General Microbiological Culture Collection Center (CGMCC) under the accession number CGMCC No. 10044. This strain is routinely cultured in Luria-Bertani (LB) medium at 37°C.

### Cucumber plug seedling

Seeds of the cucumber (*Cucumis sativus*, Zhongnong No.6, China) in this experiment were bred and obtained from the Institute of Vegetable and Flower, Chinese Academy of Agricultural Sciences (CAAS). Cucumber seeds were surface sterilized by soaking in 5% sodium hypochlorite for 15 min and 70% ethanol for 1.5 min and then washed three times with sterile water. After water absorption for 6 h, the seeds were put into the germinator and the growth condition was set at 29°/25°C (day/night) and 80% of humidity until the seeds were germinated. These germinated seeds were sown into the 72-cell plug tray, which was filled with substrates consisting of peat moss, vermiculite, and perlite (3:1:1, v/v/v), in the green house of the Institute of Vegetable and Flower, CAAS. For treatment of *B. amyloliquefaciens* L-S60, bacterial cells was inoculated in 500 mL flask with 300 mL LB broth under the shaking condition until the culture density reached 10^9^ cfu/mL. After that, the L-S60 culture was diluted to 10^7^ cfu/mL with sterile water. 1 L of the substrate was mixed with 1 L of the diluted *B. amyloliquefaciens* L-S60 culture to reach a final concentration of 10^7^ cfu/g substrate. For the control group, the substrate was mixed with the same volume of sterile water (3 replicates per each treatment). Substrate samples and seedlings were collected at 5, 10, 15, 20, and 25 days after sprout from each group. The original substrate samples of each group were also collected. At each time points 3 substrate samples from 3 different plugs and 18 randomly picked seedlings (6 seedlings per plug) were collected. The shoot height, stem diameter, leaf area, fresh weight and minerals of seedlings were measured. Specifically, shoot height was measured from base of the shoot to the tip of the tallest leaf; stem diameter was measured with a calipers just below the cotyledon scar; leaf area was determined using a digital scanner with area calculation software (Microtex, Taiwan); the fresh weight was measured by an electronic balance (Mettler, OH, USA). Substrate samples from each plug were separated into two parts: one was dried for measurements of major available minerals and the other was stored at −80°C until the subsequent DNA extraction. No chemical fertilizers and pesticides were applied in this experiment.

### Measurements of available minerals

Nitrogen, phosphorus and potassium are three most common and important major available minerals in the soil for plant growth. For measurement of the available nitrate in the substrate, we used a published alkali hydrolysis method (Greenfield, 2001). Briefly, 2 g of the dried substrate sample was weighed, mixed with 20 mL of 5 M NaOH in a 250 mL flask, which was connected to a glass reflux condenser in a oil bath, and maintained at 110°C for 24 h. After that, the flask was cooled down. The supernatant with available nitrogen was collected after centrifugation at 5,000 g for 5 min. Alkali supernatants were chilled whilst ice-cold 6 M HCl was slowly added, until a pH of 6.5 was reached. Neutralized hydrolysates were adjusted to the volume of 100 mL and aliquots were taken out for determination of available nitrogen by the published method (Bremner, 1965). The Agilent 730-ES inductively coupled plasma optical emission spectroscopy (ICP-OES) with CCD detection (Agilent, CA, USA) was applied to analyze the available phosphorus and potassium concentrations in the substrate according to the manufacture's protocol (Guidance IO-035 from Agilent). To briefly explain, 0.25 g of dried substrate sample was weighed and added into the microwave digestion vessels. 9 mL of 10 M HNO_3_ and 3 mL of 10 M HCl were further added. After digestion, the solution was cooled, centrifuged at 5,000 g for 30 min, and transferred into a 50 mL flask. Each solution was diluted to a volume of 20 mL with deionized water. After calibration, the phosphorus and potassium levels were measured via absorption under the wavelength of 178 and 766 nm, respectively. For each sample, duplicate measurements were carried out.

### DNA extraction

Substrate DNA was extracted by using the E.Z.N.A.® soil DNA kit (OMEGA, GA, USA) according to the manufacturer's instructions. The concentration and purity of the genomic DNA was evaluated by a NanoDrop 2000 spectrophotometer (Thermo, MA, USA), using an A260/A280 ratio between 1.8 and 2.0 as the criteria for quality control.

### Amplification of V3-V4 hypervariable region of the 16S rRNA genes and barcoded sequencing

The V3-V4 hypervariable region of the 16S rRNA genes were amplified by using the universal primers (340F:5′-CCTACGGGNBGCASCAG-3′ and 805R:5′-GACTACNVGGGTATCTAATCC-3′). Thermal cycling consisted of initial denaturation at 95°C for 3 min, followed by 30 cycles of denaturation at 95°C for 30 s, annealing at 50°C for 30 s, and elongation at 72°C for 60 s, and finally, at 72°C for 7 min for completion. PCR amplicons from 36 samples (12 samples × 3 replicates) were purified individually with GeneJET Gel Extraction Kit (QIAGEN, Germany) and then pooled together with equal amounts. The sequencing library was generated using KAPA Library Preparation Kit (Kapa, MA, USA) following the manufacturer's instructions and quantified by Agilent Bioanalyzer 2100 system. Finally, the quantified library was sequenced on the HiSeq2500 platform (Illumina, CA, USA), with the generation of 2 × 250 base pairs (PE250).

### Data analysis

Paired-end reads from the sequenced amplicons were stitched using FLASH (Fast Length Adjustment of SHort reads), a fast and accurate analysis tool designed to merge paired-end reads when there are overlaps between reads (Caporaso et al., 2010; Magoc and Salzberg, 2011). Meanwhile, the reads, which could not be assembled, were discarded. Paired-end reads were assigned to each sample according to the unique barcodes. The raw sequencing reads were quality-filtered using QIIME 1.9.1 software package (Quantitative Insights Into Microbial Ecology; Caporaso et al., 2010).

Operational taxonomic units (OTUs) were clustered with 97% similarity, using the subsampled open-reference-based OTU-picking workflow in QIIME based on UCLUST. Then, chimeric sequences were identified using UCHIME, and subsequently filtered out. The taxonomy of the generated OTUs was analyzed by RDP Classifier (Wang et al., 2007) against the Silva rRNA gene database with the confidence threshold of 80%. All samples were normalized at the same sequence depth and the OTUs were used to calculate alpha-diversity indices (Chao1, Shannon and Simpson) by using in-house Perl Scripts. Beta-diversity indices between samples were determined based on weighted and unweighted UniFrac distance matrices (Lozupone and Knight, 2005), which were also applied for Principal coordinates analysis (PCoA). A two-dimensional plane determined by PCoA was used to determine whether communities with similar characteristics tend to cluster together.

## Results

### Treatment by *B. amyloliquefaciens* L-S60 promoted the growth and development of cucumber seedlings

**Figure 1:**
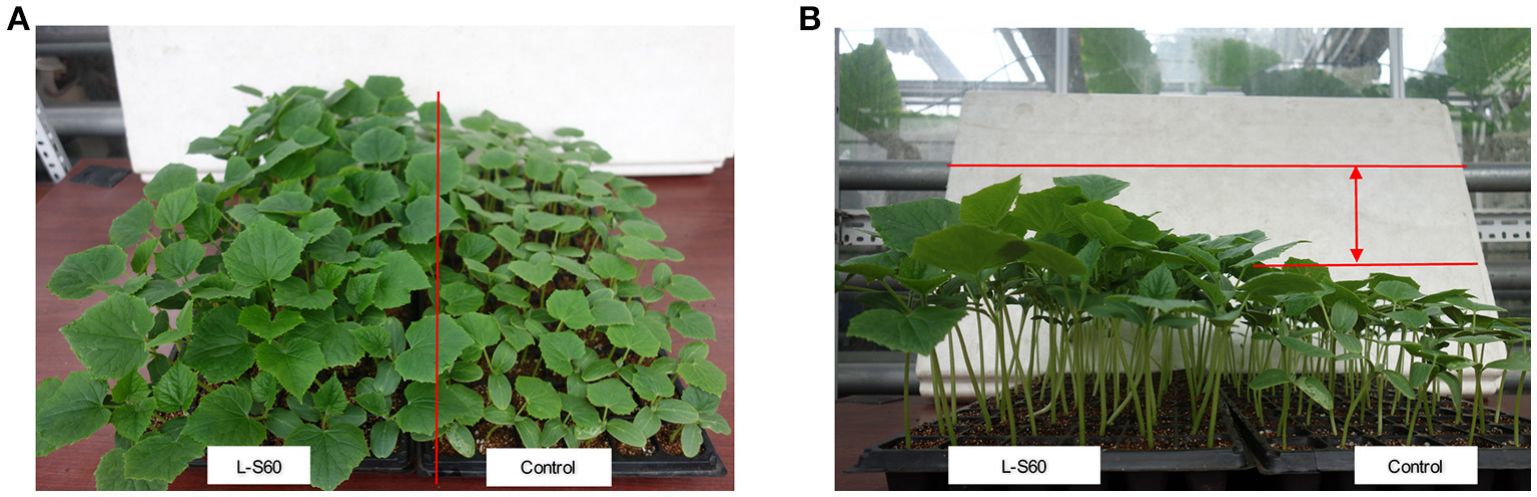
Views of cucumber seedlings grown in plug trays with different treatments. L-S60 represents seedlings treated by *B. amyloliquefaciens* L-S60; control represents the seedlings without L-S60 treatment. **(A)** Aerial view of the seedlings with different treatments; **(B)** Side view of the seedlings with different treatments.

**Figure 2:**
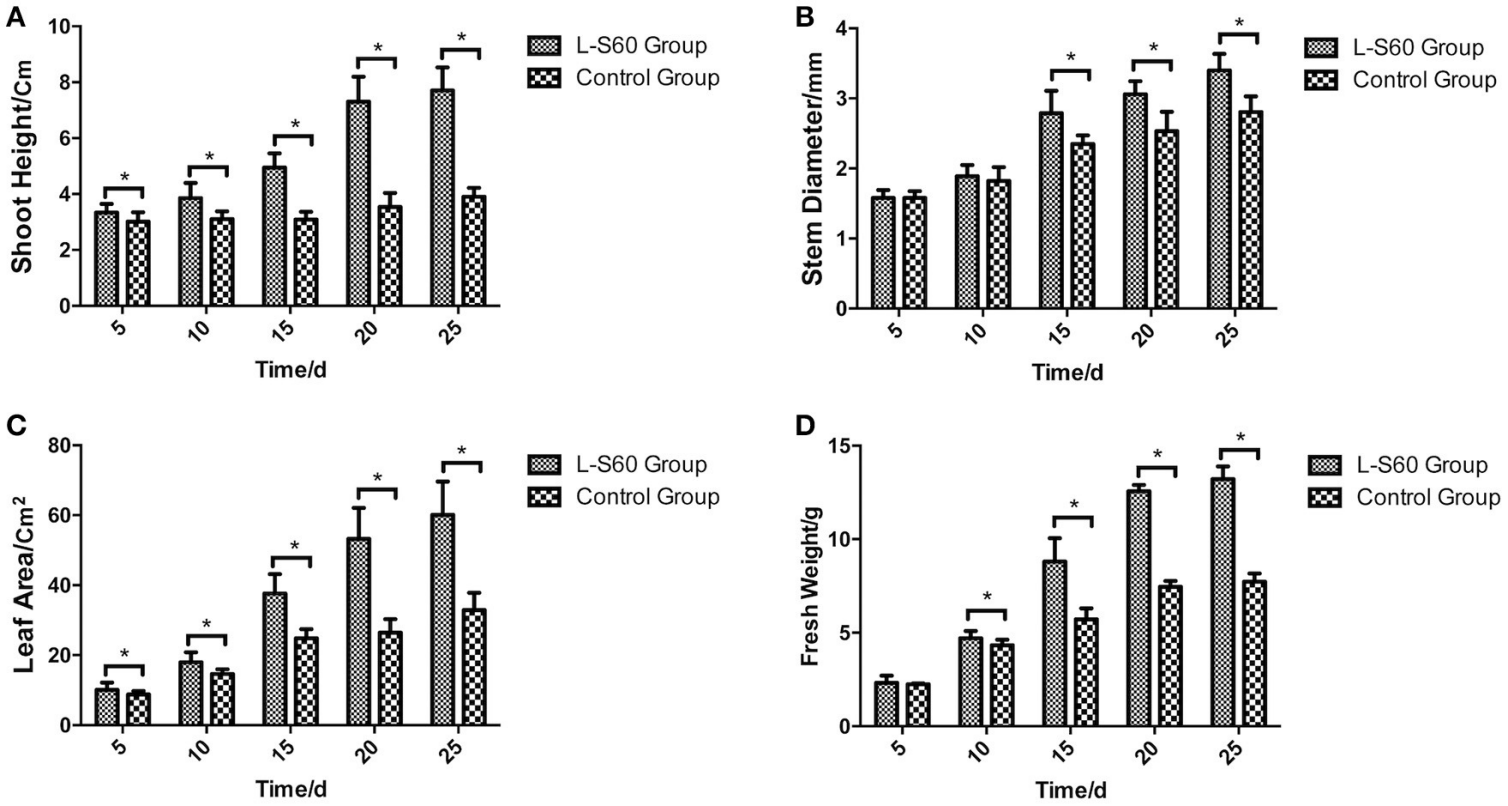
Physical indexes of cucumber seedlings grown in plug trays. L-S60 group represents seedlings treated by *B. amyloliquefaciens* L-S60; control group represents the seedlings without *B. amyloliquefaciens* L-S60 treatment. The *x*-axis represents the dates of seedling sample collection, Numbers “5–25” represent day 5, 10, 15, 20, and 25 of seedling collection after sprout. The *y*-axis represents: **(A)** height of the seedling, **(B)** stem diameter of the seedling, **(C)** leaf area of the seedling, and **(D)** fresh weight of the seedling, respectively. Values are mean ± SD (*n* = 15), with significant differences by Student's *t*-test; “^*^” indicates significant difference (*p* < 0.05).

A representative picture demonstrating the impact of L-S60 treatment on the growth of the cumber seedlings was shown in Figure 1. From the aerial view (Figure 1A) and side view (Figure 1B), we observed that the seedlings from the L-S60 treatment group showed larger leaf area and taller shot height than those in the control group. There were significant differences (*p* < 0.05) in shoot height, stem diameter, and leaf area between the treatment and the control groups (Figures 2A–C). The seedlings also showed a higher fresh weight in the L-S60 treatment group than in the control group (*p* < 0.05, Figure 2D). Average increases in shoot height, stem diameter, leaf area, and fresh weight of seedlings in the treatment group over the control group were 59.6 ± 42.6%, 12.9 ± 10.1%, 54.8 ± 37.1%, and 41.0 ± 32.7%, respectively. The maximum increases in seedling shoot height, stem diameter, leaf area, and fresh weight during the entire plug process were 106.3% (at day 20), 21.1% (at day 25), 101.4% (at day 20), and 71.0% (at day 25), respectively (Figure 2). Our data indicated that L-S60 showed strong growth promoting activities on the cucumber seedlings.

### L-S60 application did not alter the overall diversity of the rhizosphere microbial community

The rhizosphere micrbiota is known to greatly influence plant growth and health. We next investigated how application of L-S60 may have impacted the overall diversity of the rhizosphere bacterial community during cucumber plug seedling. Rhizosphere soil samples were collected periodically and total bacterial DNAs were extracted from those samples as described in the methods and stored at −80°C. Next, the hypervariable region of the 16S rRNA genes was amplified by PCR and sequenced using next generation high-throughput sequencing to characterize the overall diversity of the soil microbiota during the plug seedling. A total of 3,846,614 sequence reads of the V3-V4 region of the 16S rRNA genes were obtained from 36 substrate samples (12 samples × 3 replicates), with an average of 106,850 pair-end reads for each sample and a median read length of 447-bp. Interestingly, no significant difference in either the abundance (Chao1 index) or diversity index (Simpson and Shannon index) was seen between the L-S60 treatment group and the control group during the entire experimental period (Table 1). This indicated that application of L-S60 did not alter the overall diversity of the rhizosphere bacterial community.

**Table 1:** Alpha abundance and diversity indexes of different samples from L-S60 treatment group and the control group.

**Sample**	**Goods Coverage**	**Chao1**	**Shannon**	**Simpson**	**Observed Species**
0-60-a	0.99	5, 006.38	8.29	0.99	3623
0-60-b	0.99	5, 324.27	8.40	0.99	3506
0-60-c	0.99	4, 369.63	8.21	0.99	3140
0-ck-a	0.99	4, 957.53	8.54	0.99	3533
0-ck-b	0.99	4, 661.25	8.64	0.99	3312
0-ck-c	0.99	4, 563.51	8.64	0.99	3397
5-60-a	0.98	4, 998.73	7.74	0.98	2770
5-60-b	0.98	4, 902.75	7.88	0.98	2752
5-60-c	0.99	5, 194.01	7.65	0.98	3010
5-ck-a	0.98	5, 536.67	8.02	0.97	3605
5-ck-b	0.98	6, 081.98	7.98	0.97	3606
5-ck-c	0.98	5, 608.58	7.92	0.97	3434
10-60-a	0.98	7, 213.24	7.87	0.98	3890
10-60-b	0.99	6, 813.88	8.18	0.98	4034
10-60-c	0.99	6, 888.85	8.37	0.99	4056
10-ck-a	0.98	6, 392.90	8.56	0.99	4192
10-ck-b	0.98	8, 073.53	8.42	0.98	4991
10-ck-c	0.98	11, 294.62	8.48	0.98	5920
15-60-a	0.97	13, 017.35	8.63	0.98	6548
15-60-b	0.97	14, 221.85	8.79	0.99	7250
15-60-c	0.96	14, 446.15	8.82	0.99	7306
15-ck-a	0.97	13, 562.47	8.73	0.98	7067
15-ck-b	0.97	13, 047.92	8.81	0.99	6655
15-ck-c	0.97	10, 371.99	8.55	0.98	5247
20-60-a	0.96	13, 952.91	9.03	0.99	7178
20-60-b	0.96	15, 310.81	9.13	0.99	7718
20-60-c	0.97	12, 422.44	8.77	0.99	6166
20-ck-a	0.98	12, 248.15	8.69	0.98	6128
20-ck-b	0.97	12, 904.04	8.96	0.99	7011
20-ck-c	0.97	11, 614.62	8.79	0.99	6116
25-60-a	0.97	13, 650.51	8.80	0.99	6871
25-60-b	0.97	12, 782.57	8.93	0.99	6264
25-60-c	0.96	14, 694.55	9.11	0.99	7664
25-ck-a	0.97	13, 609.02	9.25	0.99	7106
25-ck-b	0.97	9, 218.58	9.13	0.99	5138
25-ck-c	0.96	13, 302.07	9.15	0.99	6525

*Number “0” represents the substrate samples collected on the day of sowing, numbers “5–25” mean the substrate samples collected on day 5, 10, 15, 20, and 25 after sprout. The number “60” represents samples collected from L-S60 treatment group; “ck” represents the samples collected from the control group without L-S60 treatment. The letters “a–c” represent three replicates at different time points. Chao1 is an index estimating the abundance of the bacterial community; Shannon and Simpsom are two different indexes estimating the diversity of the bacterial community*.

### L-S60 application altered rhizosphere bacterial communities at the phylum level

**Figure 3:**
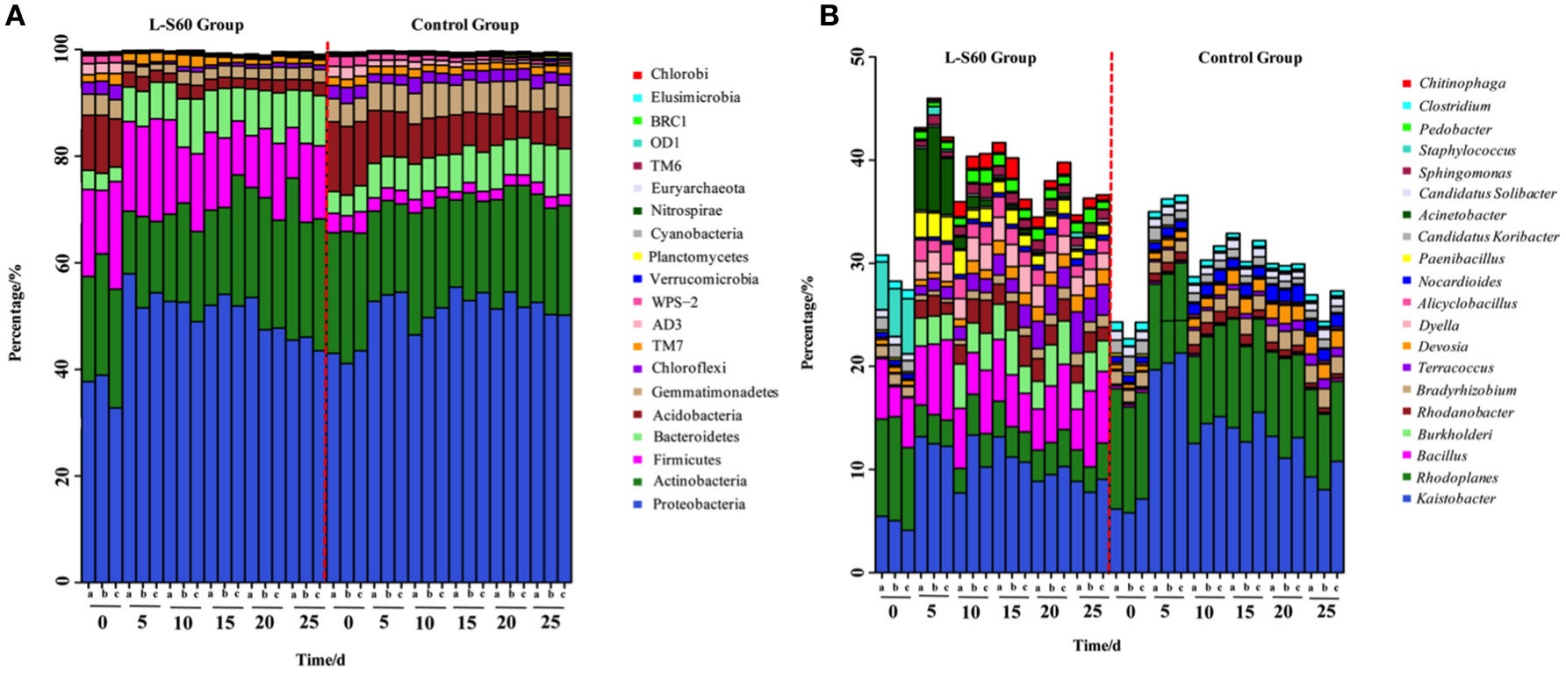
**(A)** Relative frequency of bacterial community on the phylum level in L-S60 treatment group and the control group. L-S60 group represents seedlings treated by *B. amyloliquefaciens* L-S60; control group represents the seedlings without *B. amyloliquefaciens* L-S60 treatment. The *x*-axis represents the seedling sample collection dates, Number “0” represents the substrate samples collected on the day of sowing, numbers “5–25” mean the substrate samples collected on day 5, 10, 15, 20, and 25 after sprout. The letters “a–c” represents three replicates at different time points. The *y*-axis means the relative frequencies of bacterial communities on the phylum level. **(B)** Relative frequency of the bacterial community on the genus level present in L-S60 treatment group and the control group. L-S60 Group represents seedlings treated by *B. amyloliquefaciens* L-S60; control group represents the seedlings without *B. amyloliquefaciens* L-S60 treatment. The *x*-axis represents the seedling sample collection dates, Number “0” represents the substrate samples collected on the day of sowing, numbers “5–25” mean the substrate samples collected on day 5, 10, 15, 20, and 25 after sprout. The letters “a–c” represent three replicates at different time points. The *y*-axis means the relative frequencies of the top 20 genera.

Based on the sequencing results of the 16S rRNA genes, most of the OTUs could be classified into 20 main phyla, which cover more than 99% of the total sequence reads, The most abundant phylum was found to be proteobacteria, followed by actinobacteria in both the treatment and control groups (Figure 3A). Although proteobacteria and actinobacteria were among the most abundant phyla found in all substrate samples, L-S60 treatment showed no significant impact on the abundance of these two phyla when compared to the control group (Figure 3A).

One observed impact by L-S60 inoculation was that the ratio of acidobacteria decreased dramatically from 10.1 ± 0.94% in the initial inoculation to 2.1 ± 0.25% at 25 days after spout in the treatment group, while in the control group the decrease was relatively modest (from 12.6 ± 0.56 to 6.2 ± 0.50%) (Figure 3A). Another impact by L-S60 application that we observed was on gemmatimonadetes, whose ratio also dropped from 3.8 ± 0.20% in the initial inoculation to 2.4 ± 0.14% at 25 days after sprout upon the treatment of L-S60 (Figure 3A). In contrast, the ratio of gemmatimonadetes in the control group showed an increase from 4.3 ± 0.05 to 5.1 ± 0.83% in the same period (Figure 3A). Finally, upon treatment of L-S60, the ratio of the third most abundant phylum firmicutes decreased from 16.2 ± 4.1 to 12.6 ± 2.8% when comparing the initial substrate sample to the sample collected 25 days after sprout, and both ratios were much higher than those in the control group (decreased from 3.5 ± 0.56 to 2.1 ± 0.11%). However, we suspected that this could be due to the application of L-S60, which itself is a firmicute.

Interestingly, we also noticed that the bacterial composition in the samples collected 5 days after sprout showed significant differences from the initial inoculation in both the control and the treatment groups. After 5 days, the structures of the bacterial communities seemed to stabilize and showed no significant difference to the end of this seedling process (Figure 3A). Since this happened in both the treatment and the control groups, it may be an indication of evolving microbial community during the establishment of rhizosphere microbial communities. Overall, our results suggested that application of L-S60 impacted the composition of the rhizosphere bacterial community at the phylum level, most notably in acidobacteria and gemmatimonadetes.

### L-S60 treatment altered the composition of the rhizosphere bacterial communities at the genus level

**Figure 4:**
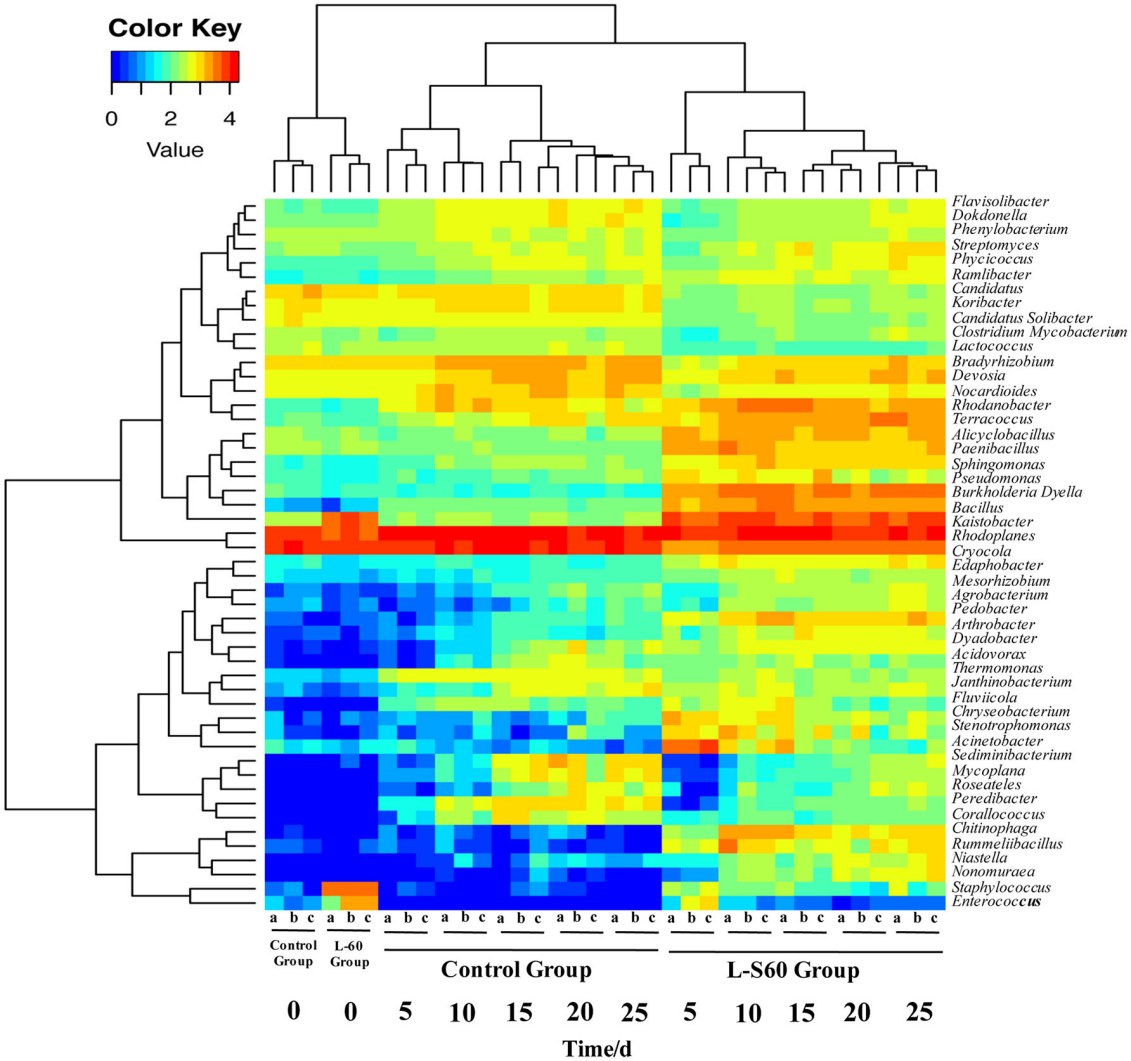
A heat map showing the bacterial community structure and composition of the top 50 genera in treated and untreated samples. L-S60 group represents seedlings treated by *B. amyloliquefaciens* L-S60; control group represents the seedlings without *B. amyloliquefaciens* L-S60 treatment. The *x*-axis represents the seedling sample collection dates. Number “0” represents the substrate samples collected on the day of sowing, numbers “5–25” mean the substrate samples collected on day 5, 10, 15, 20, and 25 after sprout. The letters “a–c” represent three replicates at different time points.

**Figure 5:**
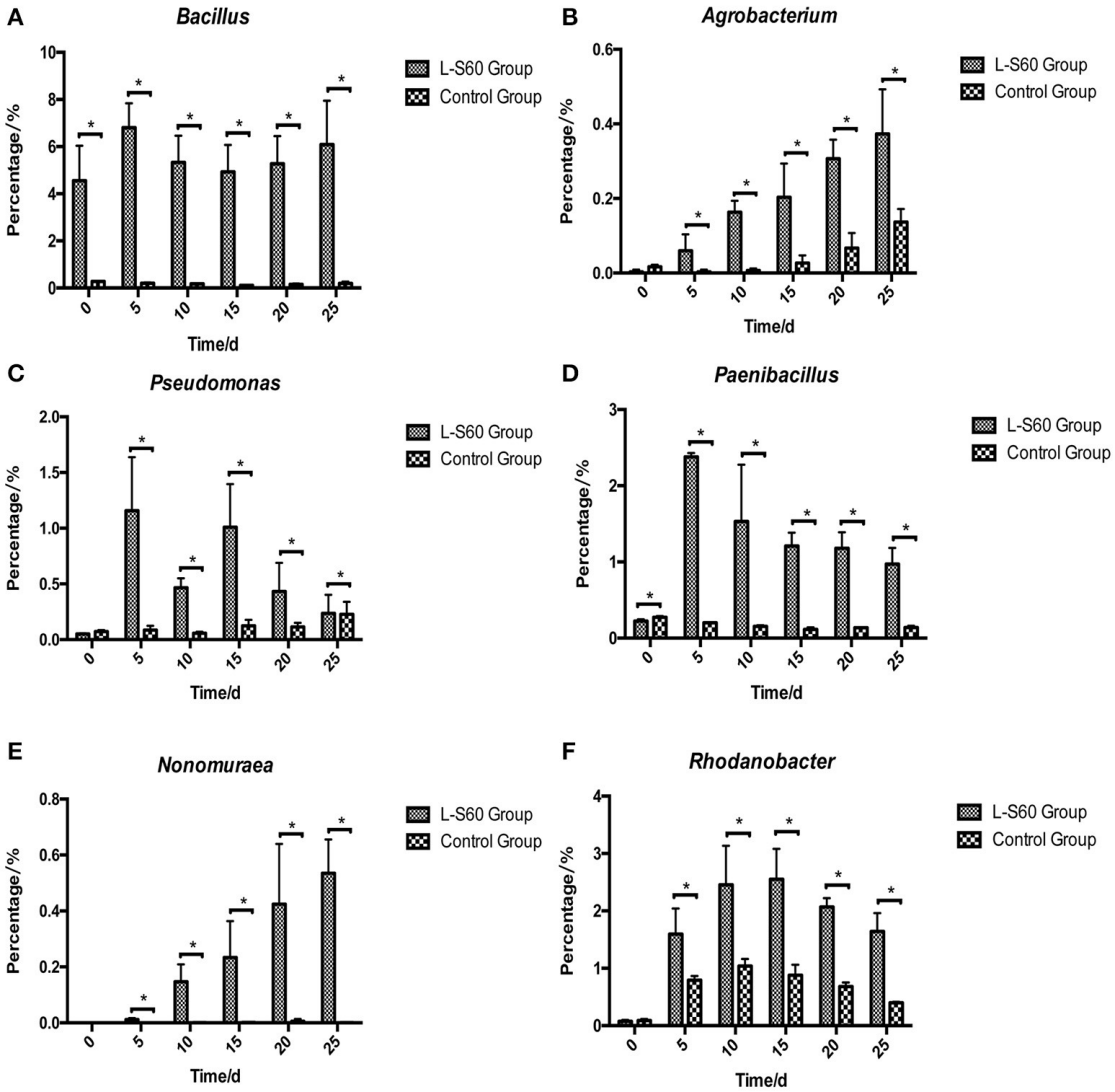
Percentages of six beneficial genera were higher in L-S60 treatment group than in the control group. L-S60 Group represents seedlings treated by *B. amyloliquefaciens* L-S60; control group represents the seedlings without *B. amyloliquefaciens* L-S60 treatment. The x-axis represents collection dates of seedling substrate samples. Number “0” represents the substrate sample collected on the day of sowing, numbers “5–25” represents the seedling substrate samples collected on day 5, 10, 15, 20, and 25 after sprout. The y-axis represents **(A)** percentage of *Bacillus*, **(B)** percentage of *Agrobacterium*, **(C)** percentage of *Pseudomonas*, **(D)** percentage of *Paenibacillus*, **(E)** percentage of *Nonomuraea*, and **(F)** percentage of *Rhodanobacter*. Values are mean ± SD (*n* = 3), with significant differences by Student's *t*-test, “^*^” indicates significant difference (*p* < 0.05).

At the genus level, top 50 genera detected in all samples were used to analyze the impact of the L-S60 treatment on rhizosphere microbiota during the seedling process (Table 2). In order to clearly outline the differences and changes between the L-S60 treatment group and the control group, heat map was generated based on the top 50 genera (Figure 4). *Kaistobacter* was the most abundant genus in all samples in both the treatment and the control groups. *Rhodoplanes* and *Bacillus* were ranked second and third in both the control and treatment groups, respectively (Figures 3B, 4). Again, the higher percentage of *Bacillus* in the treatment group might be caused by the treatment of L-S60 at the beginning of the experiment. Among the top 50 genera, the percentages of some bacterial species, such as *Bacillus, Rhodanobacter, Paenibacillus, Pseudomonas, Nonomuraea*, and *Agrobacterium*, which are known to have great impacts on the soil nutritional composition, mineral metabolism, and antibiotic production, were significantly higher in the L-S60 treatment group than in the control group (Figure 5). The differences became very significant after 5 days and stayed that way toward the remaining of the seedling process (Figure 5).

**Table 2:** A list of the top 50 abundant genera in L-S60 treatment group and the control group.

**Taxon**	**L-S60 Group(%)**	**Control Group(%)**	***p*-value**
*Kaistobacter*	0.096188	0.127865	0.025974
*Bacillus*	0.054951	0.00187	0.000999
*Rhodoplanes*	0.040773	0.090079	0.000999
*Burkholderia*	0.027572	0.000629	0.000999
*Rhodanobacter*	0.017316	0.00646	0.000999
*Terracoccus*	0.01667	0.004667	0.000999
*Dyella*	0.016643	0.001161	0.000999
*Alicyclobacillus*	0.013595	0.00163	0.000999
*Acinetobacter*	0.013339	0.000221	0.000999
*Paenibacillus*	0.01249	0.001695	0.000999
*Devosia*	0.009974	0.01104	0.474525
*Staphylococcus*	0.0096	0.000026	0.000999
*Bradyrhizobium*	0.008771	0.014643	0.000999
*Pedobacter*	0.007701	0.000397	0.000999
*Chitinophaga*	0.007488	0.000046	0.000999
*Sphingomonas*	0.007407	0.00135	0.000999
*Stenotrophomonas*	0.006135	0.000321	0.000999
*Rummeliibacillus*	0.005943	0.000038	0.000999
*Pseudomonas*	0.005584	0.001136	0.000999
*Chryseobacterium*	0.005418	0.000279	0.000999
*Cryocola*	0.004657	0.000463	0.000999
*Streptomyces*	0.00438	0.002935	0.037962
*Nocardioides*	0.004271	0.010179	0.000999
*Enterococcus*	0.003704	0.000021	0.000999
*Candidatus Koribacter*	0.00351	0.008963	0.000999
*Dyadobacter*	0.003364	0.001581	0.01998
*Arthrobacter*	0.003272	0.000308	0.000999
*Phycicoccus*	0.003155	0.002878	0.653347
*Ramlibacter*	0.003139	0.001824	0.003996
*Candidatus Solibacter*	0.002918	0.007676	0.000999
*Janthinobacterium*	0.002628	0.00255	0.925075
*Phenylobacterium*	0.002593	0.003266	0.003996
*Clostridium*	0.002511	0.005742	0.000999
*Niastella*	0.002494	0.000118	0.000999
*Flavisolibacter*	0.002308	0.004317	0.000999
*Nonomuraea*	0.002253	0.000016	0.000999
*Dokdonella*	0.002233	0.003946	0.000999
*Fluviicola*	0.002193	0.00119	0.04995
*Edaphobacter*	0.001989	0.000457	0.000999
*Mesorhizobium*	0.001879	0.000646	0.001998
*Agrobacterium*	0.001849	0.00043	0.000999
*Thermomonas*	0.00183	0.003461	0.001998
*Mycobacterium*	0.001661	0.002319	0.006993
*Acidovorax*	0.001606	0.001143	0.251748
*Roseateles*	0.00118	0.001711	0.543457
*Lactococcus*	0.001075	0.002909	0.000999
*Sediminibacterium*	0.000866	0.00365	0.007992
*Mycoplana*	0.000858	0.002906	0.027972
*Peredibacter*	0.000857	0.004342	0.000999
*Corallococcus*	0.000761	0.002286	0.016983

### L-S60 treatment altered the overall cucumber rhizosphere bacterial structure

**Figure 6:**
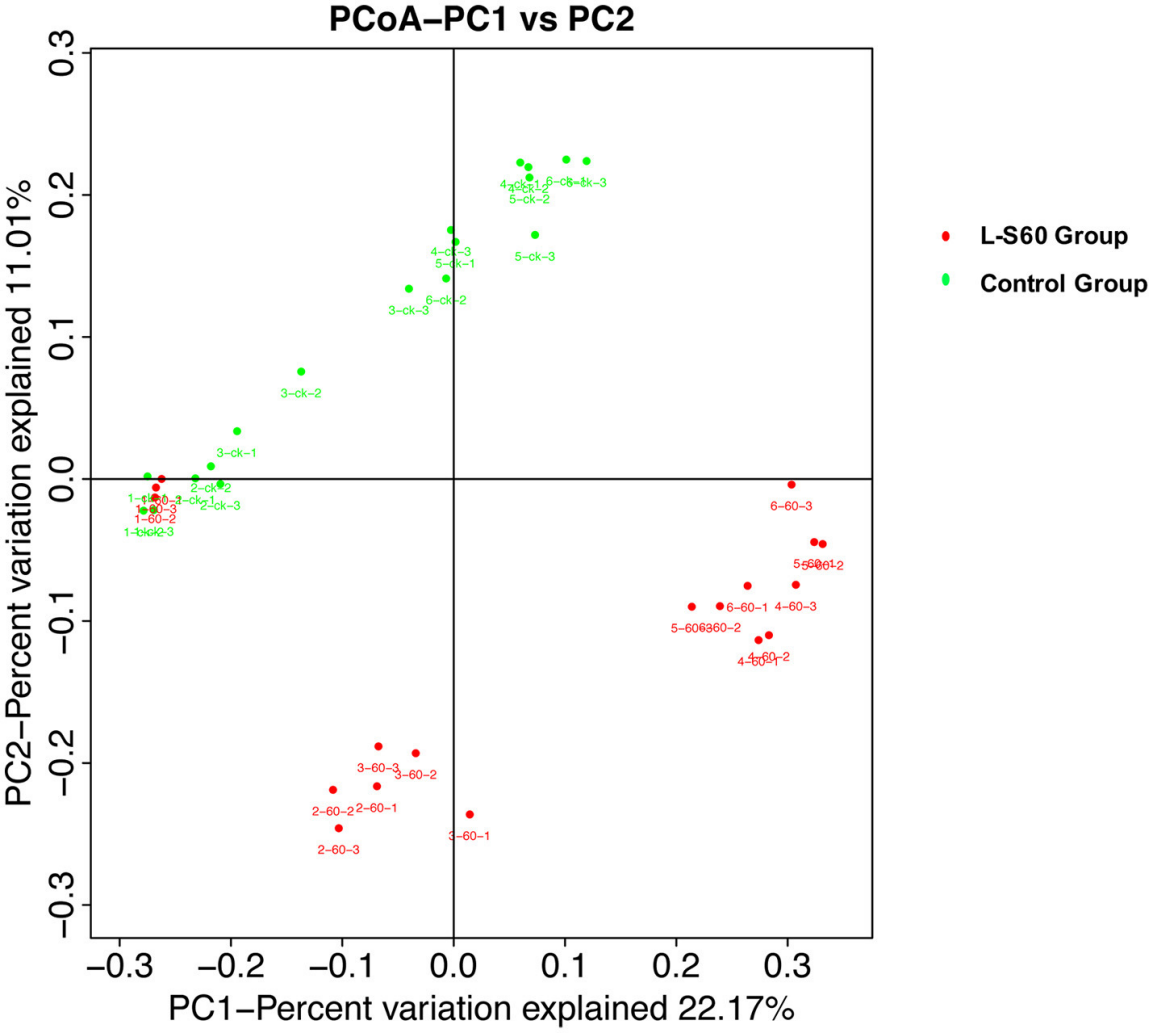
Principal component analysis of the relative abundance of OTUs. L-S60 Group represents seedlings treated by *B. amyloliquefaciens* L-S60; control group represents the seedlings without *B. amyloliquefaciens* L-S60 treatment. The PC1 represents the maximum variation factor and PC2 represents the second principal coordinate. First numbers “1–6” mean different time points for sample collection; “one” represents the day of sowing, “2–6” means day 5, 10, 15, 20 and 25 after sprout. Number “60” means the samples from L-S60 treatment group; “ck” represents the samples from uninoculation control group. Second number “1–3” means three replicates at different time points. For example, the sample “1-60-1” means the first sample collected on the day of sowing from L-S60 treatment group, “1-60-2” means the second sample collected on the day of sowing from L-S60 treatment group. Names of other samples follow the same manner.

To further investigate the soil microbiota structure between the L-S60 treatment group and the control group, principal coordinates analysis (PCoA) was performed. The result showed that there were significant differences in soil microbiota community between the L-S60 treatment group and the control group, samples in each group were clustered together into their own area (Figure 6). They accounted for 22.8 and 11.0% of the total changes. Also, in the same group, differences appeared among different time points of sampling during the whole seedling process. The microbiota structures of the first two time points were similar and the remaining ones showed the similar structure in the same group (Figure 6).

### L-S60 treatment improved the availability of major minerals in the substrate

**Figure 7:**
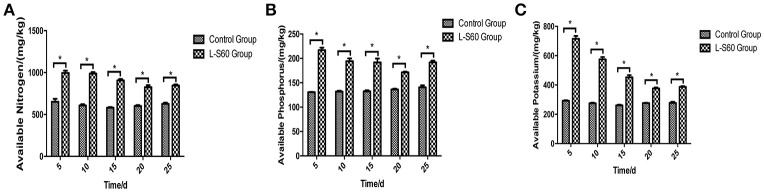
Available mineral components in the seedling substrate in L-S60 treatment and control groups. L-S60 Group represents seedlings treated by *B. amyloliquefaciens* L-S60; control group represents the seedlings without *B. amyloliquefaciens* L-S60 treatment. The x-axis represents the seedling substrate samples collection dates, Number “5–25” represents the seedling substrates collected on day 5, 10, 15, 20, and 25 after sprout. The y-axis represents **(A)** available nitrogen content, **(B)** available phosphorus content, and **(C)** available potassium content. Values are mean ± SD (*n* = 3), with significant differences by Student's *t*-test, “^*^” indicates significant difference (*p* < 0.05).

The available mineral components in the substrate were determined at different time points during the entire seedling period. Our results showed that the available nitrogen, phosphorus and potassium were significantly higher (*p* < 0.05) in the substrate sample in the group treated with L-S60 than in the control group (Figure 7). The average improvements of substrate available nitrogen, phosphorus and potassium contents in the treatment group compared with the control group at different time points were 48.7 ± 12.1, 44.1 ± 14.92, and 80.4 ± 46.4% respectively (Figure 7). The maximum improvements of available nitrogen, phosphorus and potassium contents in the substrate were 62.3% (at day 10), 66.1% (at day 5), and 144.3% (at day 5) respectively (Figure 7). The observed improvements were likely caused by different bacterial compositions in the L-S60 treated and the control groups. The available mineral components in the substrate in both groups showed a decrease trend in the 30-day cultivation period due to the absorption and utilization of those minerals by seedlings. In conclusion, our results indicated that L-S60 treatment improved the availability of essential minerals including nitrogen, phosphorus, and potassium during the seedling process.

## Discussion

*Bacillus* species are well-studied plant growth promoting rhizosphere bacteria, likely (or primarily) due to their capacities to produce antibiotics or plant hormones and to form biofilms on the root surface of the plants (Idris et al., 2007; Chen et al., 2009, 2012; Rahman et al., 2015). Furthermore, they may impact the structure of bacterial community in the plant rhizoshpere through bacteria-bacteria and bacteria-host interactions and the availability of minerals in the rhizoshpere via secreted enzymes and alteration of microbial communities. In this study, we investigated the impact of *B. amyloliquefaciens* L-S60 on rhizosphere bacterial communities during the cucumber seedling process by applying culture-independent 16S rRNA gene high-throughput sequencing. We also compared the availability of the main mineral elements in the cucumber substrates at different time points during the seedling period in both the L-S60 treated and the control groups.

### Alteration of rhizosphere microbial community

Previous studies showed that PGPR could promote plant growth by changing the micro-ecological environment in rihzosphere. In this study, we showed that L-S60 treatment altered the structure of the rihzosphere microbiome at both the phylum and genus levels (Figures 3A,B). On the phylum level, proteobacteria and actinobacteria were predominant in all samples in both the treatment and control groups, and the abundance of them varied during the growth of cucumber seedlings. Further, this variation demonstrated a similar trend from day 5 to day 25 during the seedling process in both the treatment and control groups. In contrast, there were more firmicutes in the treatment group from the beginning to the end of the experiment, which was likely due to the L-S60 usage. The level of firmicutes remained stable during the entire seedling process, indicating that L-S60 could efficiently colonize the cucumber rhizosphere. This also implies that the efficacy of L-S60 in promoting the growth of cucumber seedlings likely sustains in the whole process of plug seedling.

The newly established phylum acidobacteria showed higher percentage in the control group although they had the similar abundance at the beginning in both groups (Figure 3A). This implied that, the L-S60 application could decrease the amount of acidobacteria. Gemmatimonadetes showed a similar trend with acidobacteria at the phylum level (Figure 3A). Bacteria belonging to this phylum make up about 2% of total soil bacterial communities and it has been identified as one of the top nine phyla found in soils (Drees et al., 2006). Gemmatimonadetes in soil tends to be associated with the moisture availability since members of this phylum prefer dryer soils (Drees et al., 2006). Thus, we speculated that colonization of L-S60 in the rhizosphere of cucumber could better preserve moisture in the soil and lead to the changes of bacterial structure (e.g., decrease of gemmatimonadetes) and better growth of cucumber seedlings in the treatment group.

Another interesting observation worth noting is that most of the changes appeared within the first 5 days after L-S60 inoculation (Figure 3A). After 5 days, the structures of bacterial communities in both the control and the treatment groups seemed to stabilize and showed much less fluctuation to the end of this seedling process. This result is consistent with a previous study showing that the application of PGPR affected the rhizosphere bacterial community in early stages of plant development (Sugiyama et al., 2014). The exact mechanisms of why L-S60 primarily impacted the rhizosphere bacterial community during early stages of plant development are not clear yet. Some of the previous studies showed that the plant root exudates could alter the bacterial community. Production of the root exudates changes dramatically during the growth of the plant in that much higher amounts of exudates are released into the rhizosphere often at the initial growth phase (Baudoin et al., 2002). Thus, the changes observed in this study could also be due to that application of L-S60 impacted the secretion and composition of the cucumber root exudates at early stages of the seedling development, while minimal changes of the bacterial community from day 5 to day 25 in both the treatment and the control groups might indicate relatively stable quantity and composition of the cucumber root exudates during this period of time.

On the genus level, differences in the rhizosphere microbiome structure were also observed between the L-S60 treatment and the control groups. For example, *Paenibacillus* was present at a higher level in the treatment group (Figure 5D). It is a genus of anaerobic, endospore-forming bacteria, initially belongs to the genus *Bacillus*, and is then reclassified as a new genus (Ash et al., 1993). Some species in this genus such as *P. polymyxa* are capable of fixing nitrogen, and have been applied in agriculture and horticulture as a biofertilizer (Lal and Tabacchioni, 2009). *Rhodanobacter* is another genus of bacteria, which showed larger amounts in the substrate of the treatment group (Figure 5F). It has the capacity to perform complete denitrification in the soil (Green et al., 2012; Prakash et al., 2012). High concentrations of nitrate inhibit majority of microbes, but not *R. denitrificans*, a member of *Rhodanobacter* that can survive the nitrate rich environment and conduct denitrification, a process crucial to the ecosystem (Green et al., 2012). *Rhodanobacter* may produce more available nitrate in the soil for cucumber growth by denitrification. *Pseudomonas*, another well-studied and widely used biocontrol agent, also had a higher ratio in the L-S60 treatment group than in the control group (Figure 5C). Species in *Pseudomonas*, for instance *P. fluorescens*, have been shown to induce the plant defense capacity against certain pathogens and thus improve plant health. Also, *P. fluorescens* might inhibit the growth of other pathogenic soil microbes by scavenging iron or producing phenazine-type antibiotics or hydrogen cyanide compounds, which are antagonistic to other soil microbes (Haas and Défago, 2005).

### Improvement of plant growth conditions

PGPR promote plant growth promotion. In our study, we directly measured cucumber physiological indexes and found significant differences between the control and L-S60 treatment groups (Figure 2). Meanwhile, we also found that the available nitrogen, phosphorus and potassium in the seedling substrate of treatment group were higher than those in the control group (Figure 7). We speculated that the inoculation of L-S60 changed the structure of the microbial community of the cucumber seedling rihzosphere, especially resulting in the increase of the abundance of certain soil bacteria. Those soil bacteria could change the content of available mineral elements in the seedling substrate and improve the uptake of the elements by plants (Talboys et al., 2014). In the case of nitrogen, as we have discussed above, enrichment of certain microbial species such as *Rhodanobacter* upon treatment of L-S60 may improve utilization of nitrogen sources.

Potassium is the second major plant macronutrient after nitrogen. Potassium availability impacts the growth and development of plant (Meena et al., 2015). Moreover, potassium not only participates in nutrient transportation and uptake, but also renders plants more resistant to environmental stresses, thus leading to a higher yield and quality of crops (Pettigrew, 2008). *Agrobacterium tumefaciens* is a potassium-solubilizing bacterium, which solubilizes fixed forms of potassium and makes it available to plant by various mechanisms including acidolysis, chelation, exchange reactions, complexolysis, and production of organic acids (Meena et al., 2015). We found that the ratio of *Agrobacterium* was higher in L-S60 treatment group than in the control group (Figure 5B). Presumably, the higher presence of *Agrobacterium* will lead to more available potassium in the seedling substrate, which was we have observed in our study when we measured the potassium availability upon L-S60 treatment (Figure 7).

Phosphorus is another kind of major essential macronutrients for plant growth and development (Rodríguez and Fraga, 1999). But the concentration of phosphorus in soil that is bioavailable to plant is extremely low. Microorganisms play an important role during the phosphorus metabolism in natural environment. Several studies have shown that different kinds of bacteria could solubilize inorganic phosphate compounds into the available phosphorus, which could then be utilized by plants. These bacteria include *Pseudomonas, Bacillus, Rhizobium, Burkholderia, Achromobacter, Agrobacterium, Microccocus, Aereobacter, Flavobacterium*, and *Erwinia* (Rodríguez and Fraga, 1999). In this study, *Pseudomonas* and *Agrobacterium* were found to be present in higher percentages in the L-S60 treatment group than in the control group (Figures 5B,C). This could explain why after the inoculation of L-S60 the content of available phosphorus was higher than that in the control group in our study.

## Conclusion

In conclusion, our work showed the rhizosphere bacterial community varied significantly during the entire cucumber plug seedling upon application of the biological control agent *B. amyloliquefaciens* L-S60, in particular, the higher presence of potential PGPRs. We also observed the improvement on cucumber growth conditions by L-S60 treatment during the plug seedling. Presumably, these PGPRs can convert more mineral elements in the substrate into bioavailable forms that can be easily absorbed by seedlings. Our investigation suggests that *B. amyloliquefaciens* L-S60 may be used as an effective biological control agent in vegetable plug seedlings. In future studies, it will also be interesting to try to combine L-S60 with other potential PGPRs to form a simple integrated recipe to promote the growth of the cucumber seedlings.

## Author contributions

YQ, QS, and PL designed the experiments. YQ, QS, and YZ performed the experiments. YQ and YC analyzed the results and wrote the manuscript.

### Conflict of interest statement

The authors declare that the research was conducted in the absence of any commercial or financial relationships that could be construed as a potential conflict of interest.
